# Psoralen inhibits malignant proliferation and induces apoptosis through triggering endoplasmic reticulum stress in human SMMC7721 hepatoma cells

**DOI:** 10.1186/s40659-019-0241-8

**Published:** 2019-07-05

**Authors:** Xiaomin Wang, Peike Peng, Zhiqiang Pan, Zhaoqin Fang, Wenli Lu, Xiaomei Liu

**Affiliations:** Basic Medical School of Shanghai University of Traditional Chinese Medicine, 1200 Cailun Road, Shanghai, 201203 China

**Keywords:** Psoralen, Hepatocellular carcinoma, SMMC7721 cell, Endoplasmic reticulum stress, Apoptosis

## Abstract

**Background:**

Psoralen is a coumarin-like and coumarin-related benzofuran glycoside, which is a commonly used traditional Chinese medicine to treat patients with kidney and spleen-yang deficiency symptom. Psoralen has been reported to show estrogen-like activity, antioxidant activity, osteoblastic proliferation accelerating activity, antitumor effects and antibacterial activity. However, the antitumor mechanism of psoralen is not fully understood. This study aimed to investigate the therapeutic efficacy of psoralen in human hepatoma cell line SMMC7721 and the mechanism of antitumor effects.

**Results:**

Psoralen inhibited proliferation of SMMC7721 in a dose- and time-dependent manner, and promoted apoptosis. Further, psoralen activated the ER stress signal pathway, including the expansion of endoplasmic reticulum, increasing the mRNA levels of GRP78, DDIT3, ATF4, XBP1, GADD34 and the protein levels of GDF15, GRP78, IRE1α, XBP-1s in a time-dependent manner. Psoralen induces cell cycle arrest at G1 phase by enhancing CyclinD1 and reducing CyclinE1 expression. Moreover, TUDC couldn’t inhibit the psoralen-induced ER stress in SMMC7721 cells.

**Conclusions:**

Psoralen can inhibit the proliferation of SMMC7721 cells and induce ER stress response to induce cell apoptosis, suggesting that psoralen may represent a novel therapeutic option for the prevention and treatment hepatocellular carcinoma.

**Electronic supplementary material:**

The online version of this article (10.1186/s40659-019-0241-8) contains supplementary material, which is available to authorized users.

## Background

Hepatocellular carcinoma (HCC) is one of the most common malignant cancer worldwide and the second leading cause of cancer death for male in China, especially in China’s rural areas, and it is also considered as the sixth leading cause of cancer-related mortality for male in developed countries. According to reports, the incidence and mortality of liver cancer in China occupies for nearly 50% of the global liver cancer population [1, 2]. Unfortunately, the pathogenesis of HCC is concealed, which is difficult to find in early stage. The rapid development and high rate of postsurgical relapse is a major challenge of HCC, owing to the rapid proliferation and differentiation of hepatoma cells [3]. Thus, new antitumor agents and potential mechanisms are urgently needed to inhibit the proliferation of hepatoma cells.

**Fig. 1:**
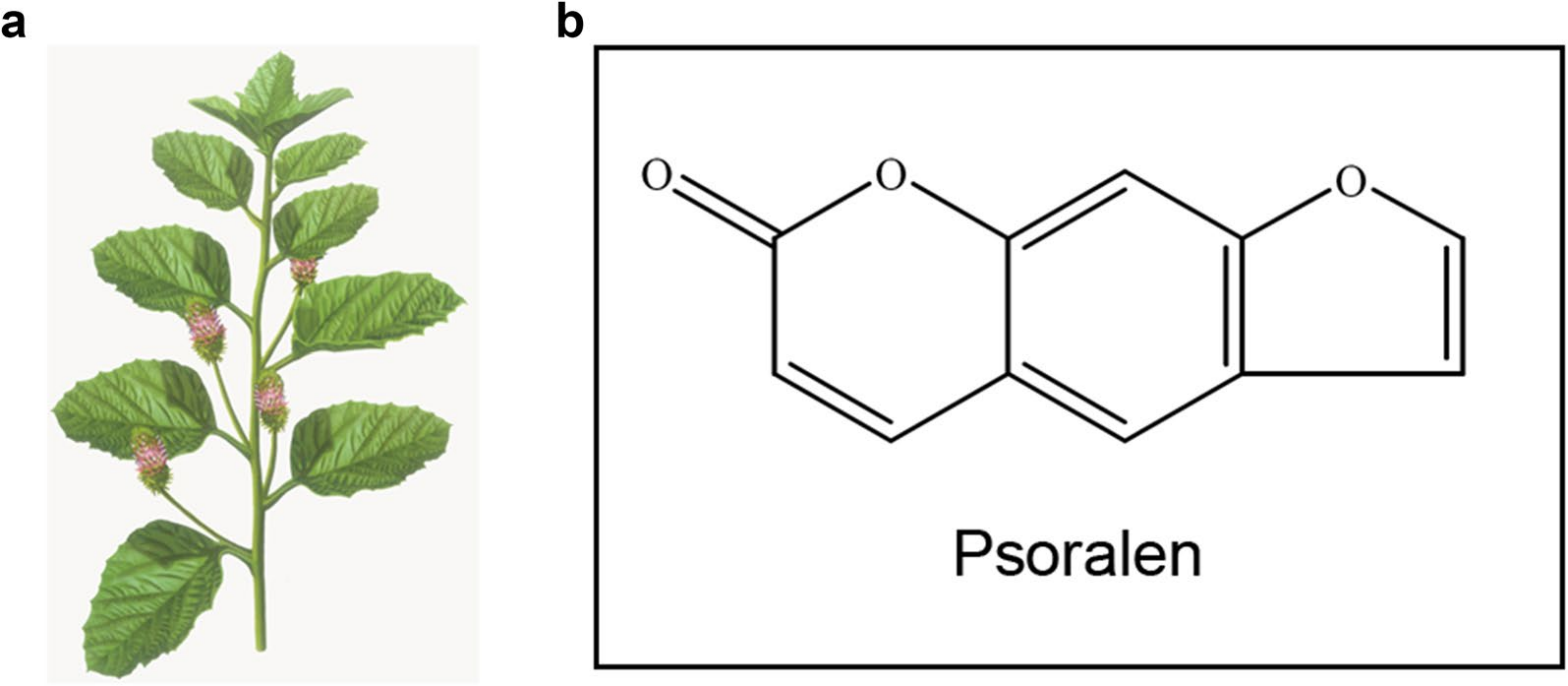
Chemical structure of psoralen. **a** The plant diagram of psoralea. **b** The chemical structure of psoralen

Sorafenib is considered to be the only effective treatment targeted drug for patients with advanced HCC, but it shows poor treatment effect for patients with extrahepatic spread or vascular invasion [4]. Therefore, screening effective anti-hepatoma drugs from traditional Chinese medicine herbs is a necessary research field. In recent decades, some effective ingredients of Chinese medicinal herbs, such as icariin, psoralen, osthole, matrine, ginsenoside Rg1/Rh2 and celastrol, were reported to inhibit the proliferation of hepatocellular carcinoma cells [5–11]. *Psoralea* species possess unique bioactive compounds with anti-cancer properties [12], and there are many tumor-suppression patents of traditional Chinese medicine which have psoralea as basic remedy [13–15]. Additionally, psoralen is the main active component of *Psoralea corylifolia* (L.) (Fig. 1a) and is used as marker to assess its quality [16, 17], which can inhibit the proliferation of adrenocortical tumors Y1 cells and pituitary tumor AtT20 cells [18]. Psoralen is a kind of furocoumarin, the molecular formula of psoralen is C_11_H_6_O_3_, with molecular weight of 186.1 and dissolved in DMSO. The chemical structure of psoralen was shown in Fig. 1b. Furthermore, psoralen plays roles in anti-tumor, anti-oxidation and reversing the multidrug resistance, and it is concerned with cell cycle, apoptosis, calcium antagonism and estrogen-like effects [19–22]. However, the effect of psoralen on HCC is still unclear.

Endoplasmic reticulum stress (ER-stress) is the basic cells response against various extracellular factors, including UV, anoxia, oxidative stress, toxic substances, nutrient deficiency and drug agonists, which will result in the unfolded proteins accumulation in the endoplasmic reticulum and lead to Ca^2+^ homeostasis imbalance. According to previous studies, three major ER-spanning transmembrane proteins, PERK (protein kinase R-like ER kinase), ATF6 (activating transcription factor 6) and IRE1 (inositol-requiring enzyme 1) subsequently drive mutually reinforcing signaling pathways to correct the protein-misfolding stress [23]. In this process, glucose-regulated protein of 78 (GRP78), also known as heavy chain-binding protein (Bip), plays an important role in detecting the accumulation of unfolded proteins in ER lumen and releasing the three sensors, so GRP78 is considered to be an ER homeostasis receptor [24].

In the early stage of ER stress, the unfolded protein response (UPR) acts to help cells to cope with the stress by attenuating protein synthesis, clearing the unfolded/misfolded proteins and increasing the capacity of the ER to fold proteins, which restore the intracellular homeostasis and protect cell functions. However, strong and sustained ER stress induced by the activation of UPR will cause imbalance of ER homeostasis. Prolonged activation of the UPR can induces apoptosis pathway following ER stress, mainly involving in the CHOP/DITT3/GADD153 gene activation pathway, JNK activation pathway, ER-specific cysteine Caspase-12 activation pathway.

In this study, we observed the proliferation inhibition effects of psoralen on hepatoma SMMC7721 cells and further investigated its relationship with ER stress. The results suggest that psoralen can induce cell cycle arrest and apoptosis by ER stress to inhibit malignant proliferation of hepatoma cells.

## Results

### Psoralen inhibits the proliferation of SMMC7721 cells

**Fig. 2:**
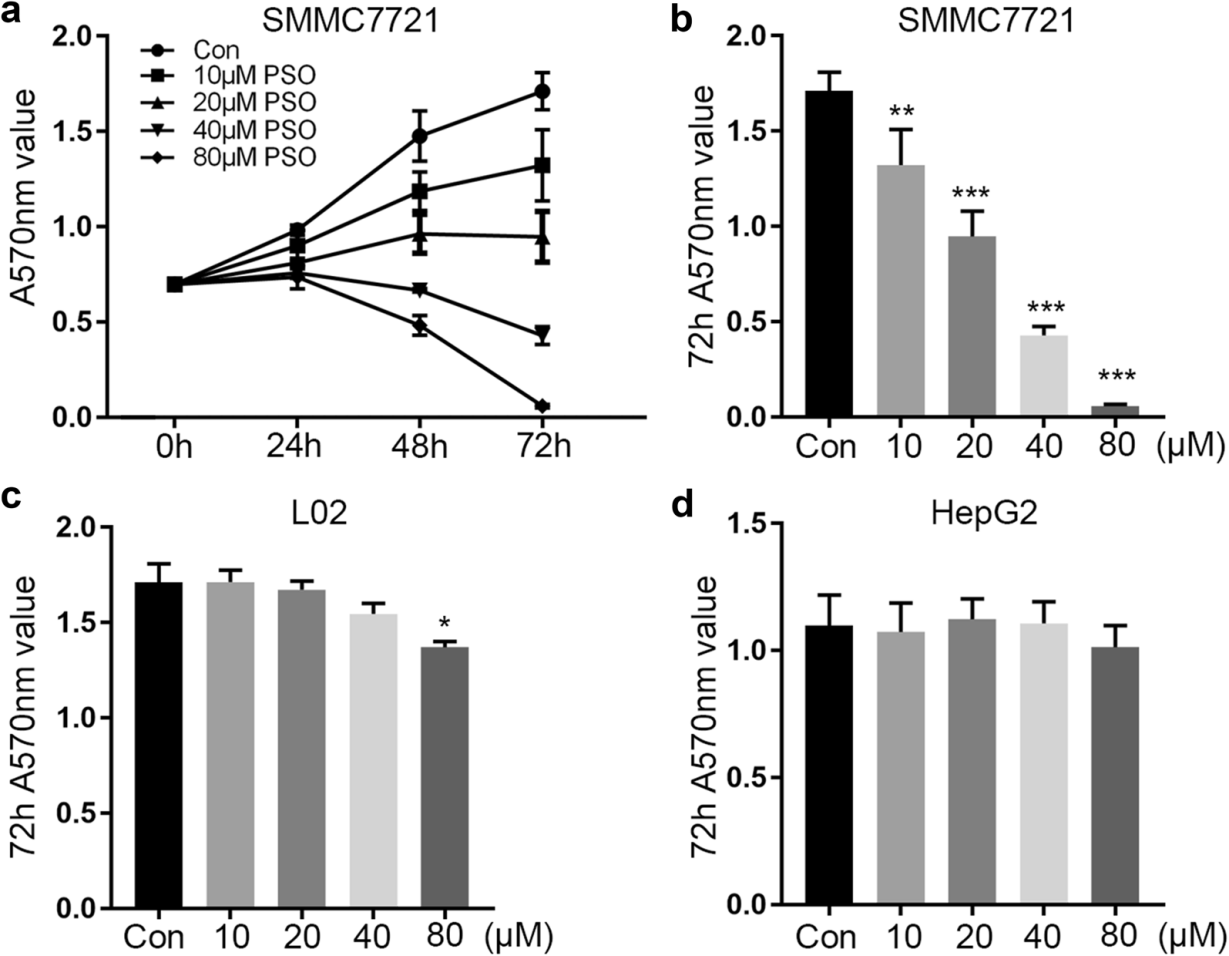
Effects of psoralen on the proliferation of SMMC7721, L02 and HepG2. **a** Different doses of psoralen inhibit the proliferation of SMMC7721 for 24–72 h. The A570 value of SMMC7721 (**b**), L02 (**c**) and HepG2 (**d**) with the treatment of psoralen in 10–80 μM for 72 h. Values are mean ± SD (n = 4) and * is means compared to the Con group, *P < 0.05; **P < 0.01; ***P < 0.001

We first examined whether psoralen was cytotoxic to hepatoma cell lines. Compared with the control group, the SMMC7721 cells treated with different dose psoralens for 24 h, 48 h and 72 h. We found that 20–80 μM psoralen inhibited cell proliferation and the inhibition rate was 17.50–25.10% in 24 h (Table 1). More than 20 μM psoralen could effectively inhibit cell proliferation in 48 h (P < 0.001) and 72 h (P < 0.001) (Table 1). In particular, more than 80 μM psoralen caused cell death (Fig. 2a, b). Next, we explored the effect of psoralen on L02 hepatocyte cell line and HepG2. We found that 10 μM, 20 μM and 40 μM PSO had little effects on the proliferation of L02, but 80 μM PSO could inhibit L02 proliferation (Fig. 2c). However, psoralen treatment showed little effect on proliferation of HepG2 cells (Fig. 2d). It is suggested that psoralen has a significant inhibitory effect on the proliferation of SMMC7721.

**Table 1:** Inhibitory effect of psoralen on proliferation of SMMC7721 hepatoma cells ($\overline{x}$ ± s, n = 3)

Dose/μM	Inhibition rate/%		
	24 h	48 h	72 h
0 (Con)	0.00 ± 2.70	0.00 ± 8.90	0.00 ± 5.70
10	8.40 ± 8.00	19.60 ± 6.90*	22.80 ± 10.9*
20	17.50 ± 1.90	34.80 ± 6.90**	44.60 ± 7.80**
40	22.90 ± 2.90	54.80 ± 0.60***	74.90 ± 2.80***
80	25.10 ± 6.40	67.30 ± 3.50***	96.60 ± 0.50***

Compared with the control group, *P < 0.05, **P < 0.01, ***P < 0.001. The data are from three independent experiments, four wells for each experiment

### Psoralen causes shrinkage and endoplasmic reticulum dilation of SMMC7721

**Fig. 3:**
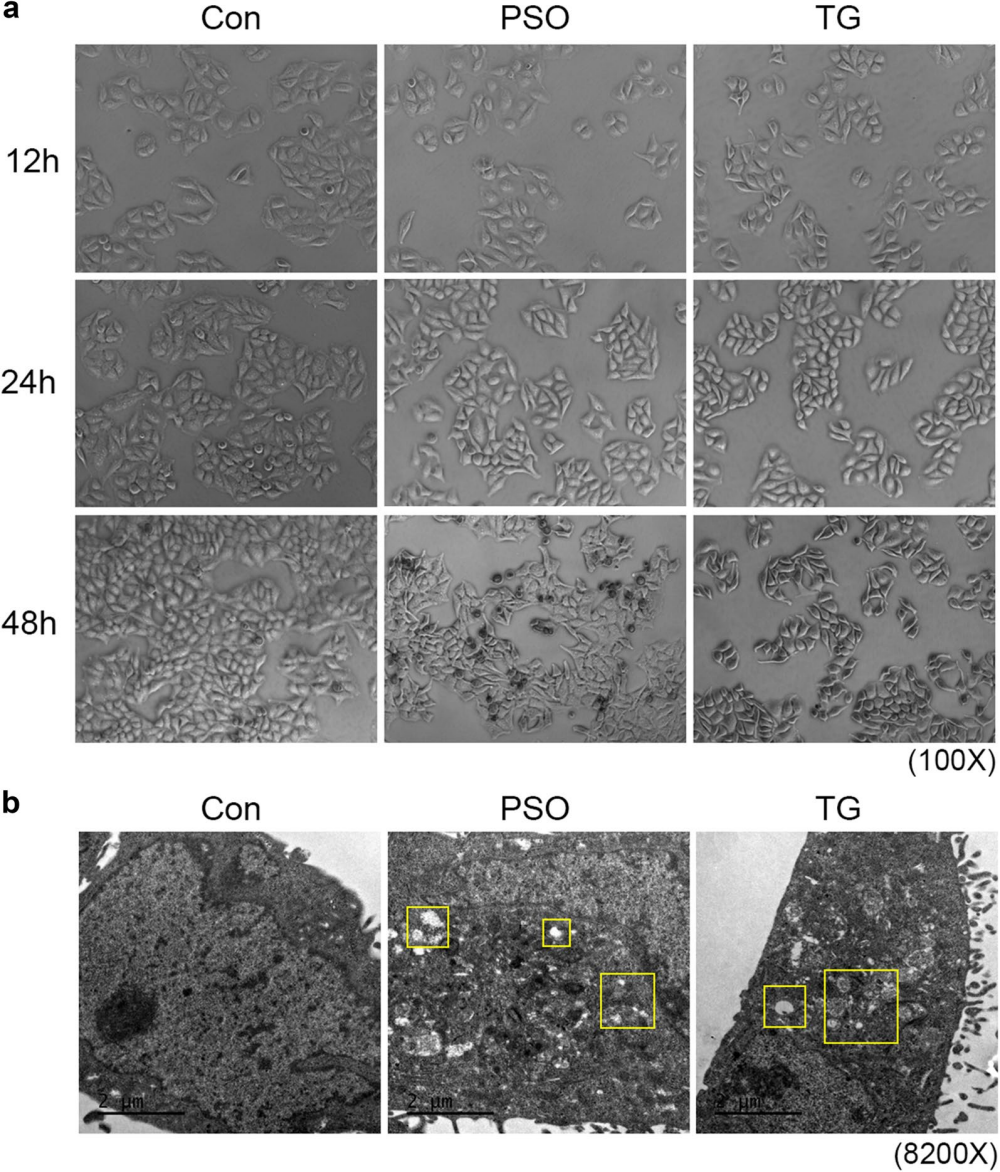
Effects of psoralen on the morphology of SMMC7721. **a** SMMC7721 cells shrunk in the dose of 40 μM psoralen or 0.1 μM thapsigargin for 12–48 h. **b** Abnormalities of endoplasmic reticulum and cytoplasmic structure in SMMC7721 in the dose of 40 μM psoralen or 0.1 μM thapsigargin for 24 h. Yellow boxes represent the abnormal region

The SMMC7721 cells were treated with 40 μM psoralen or 0.1 μM thapsigargin for 12 h, 24 h, and 48 h, respectively. The results showed that SMMC7721 cells appeared mild shrinkage and the cells gap increased. With the treatment prolonged, the SMMC7721 cells shrunk significantly, furthermore the dead and floated cells could be observed. The thapsigargin caused the cells shrinkage as a positive control, which showed significant inhibitory effect than that of psoralen (Fig. 3a). These results suggested that psoralen can induce the shrinkage and reduce adhesion of SMMC7721 cells. Next, we observed the intracellular structure of SMMC7721, which showed a large nucleus, few cytoplasm, scattered smooth endoplasmic reticulum and some mitochondria in transmission electron microscope. However, endoplasmic reticulum dilation appeared in SMMC7721 by 40 μM psoralen treatment for 24 h, and the cytoplasm presented loose and derangement distribution. Moreover, 0.1 μM thapsigargin can cause the endoplasmic reticulum dilation and cytoplasmic structure disorder (Fig. 3b). It is suggested that psoralen can cause abnormalities of endoplasmic reticulum and cytoplasmic structure in SMMC7721 hepatoma cells.

### Psoralen induces cell cycle arrest at G1 phase in SMMC7721

**Fig. 4:**
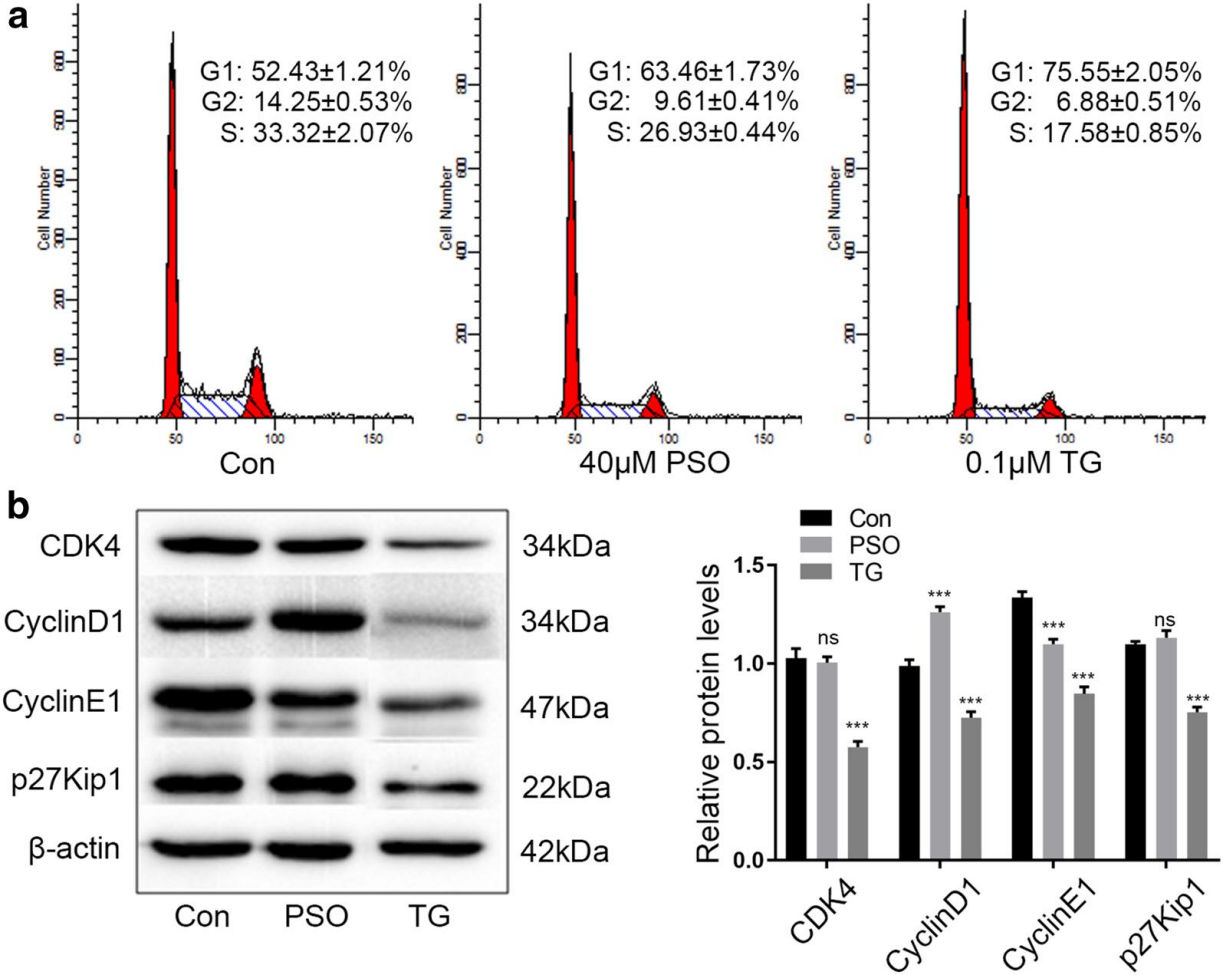
Psoralen induces cell cycle arrest at G1 phase in SMMC7721. Flow cytometry (**a**) and Western blot (**b**) assays showing the effects of psoralen and thapsigargin on cell cycle progression and expression of G1/S transition-related proteins, respectively. Relative protein levels are showed on the right. *ns* no significant; ***P < 0.001

We next evaluated whether psoralen modulated cell cycle progression to affect cell viability. PI staining analysis by flow cytometry demonstrated that 40 μM psoralen led to a significant increase in the percentage of cells at the G1 phase and a decrease in cells at the S phase. And 0.1 μM thapsigargin also caused a significant increase in the percentage of cells at the G1 phase (Fig. 4a). We also determined the effects of psoralen on the expression of G1/S transition-related cell cycle proteins. Compared with the control group, CyclinD1 protein expression was increased significantly by 40 μM psoralen, and CyclinE1 expression was suppressed significantly. However, psoralen showed little influence on the expression of CDK4 and p27Kip1 (CDKN1B). Additionally, the proteins expression of CyclinD1, CyclinE1, CDK4 and p27Kip1 were decreased significantly by 0.1 μM thapsigargin (Fig. 4b). These results suggested that psoralen could induces cell cycle arrest at the G1 phase by reducing the expression of CyclinE1 in SMMC7721, thus inhibiting the growth of hepatoma cells.

### Psoralen induces apoptosis in SMMC7721 hepatoma cells

**Fig. 5:**
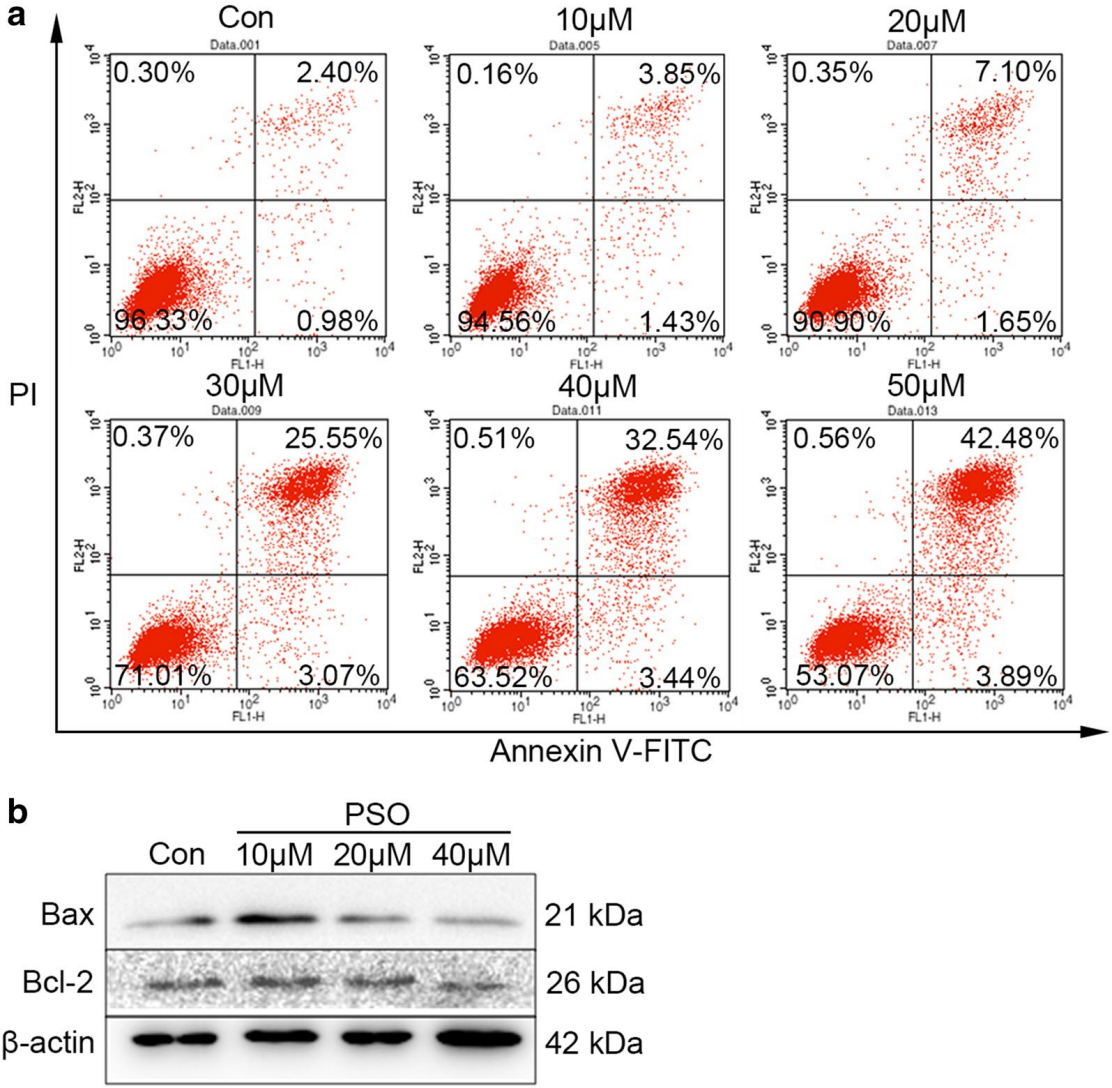
Psoralen induces apoptosis in SMMC7721 hepatoma cells. AnnexinV-PI staining (**a**) and western blot (**b**) assays showing the effects of different doses of psoralen on cell apoptosis and the expression of apoptosis-related proteins

We next determined the effects of psoralen on cellular apoptosis in SMMC7721 cells. Annexin-V staining revealed that psoralen treatment increased late apoptosis in SMMC7721 hepatoma cells and showed a significant dose-effect relationship (Fig. 5a). Western blot analysis also demonstrated that treated with 10 μM psoralens for 48 h could increase the level of Bax, and 40 μM psoralen could attenuate the expression of Bcl-2 (Fig. 5b). These results suggested that psoralen induces cell apoptosis in SMMC7721, which may be related to the inhibition of Bcl-2 protein expression.

### The effects of psoralen on ER-stress related gene expression in SMMC7721

**Fig. 6:**
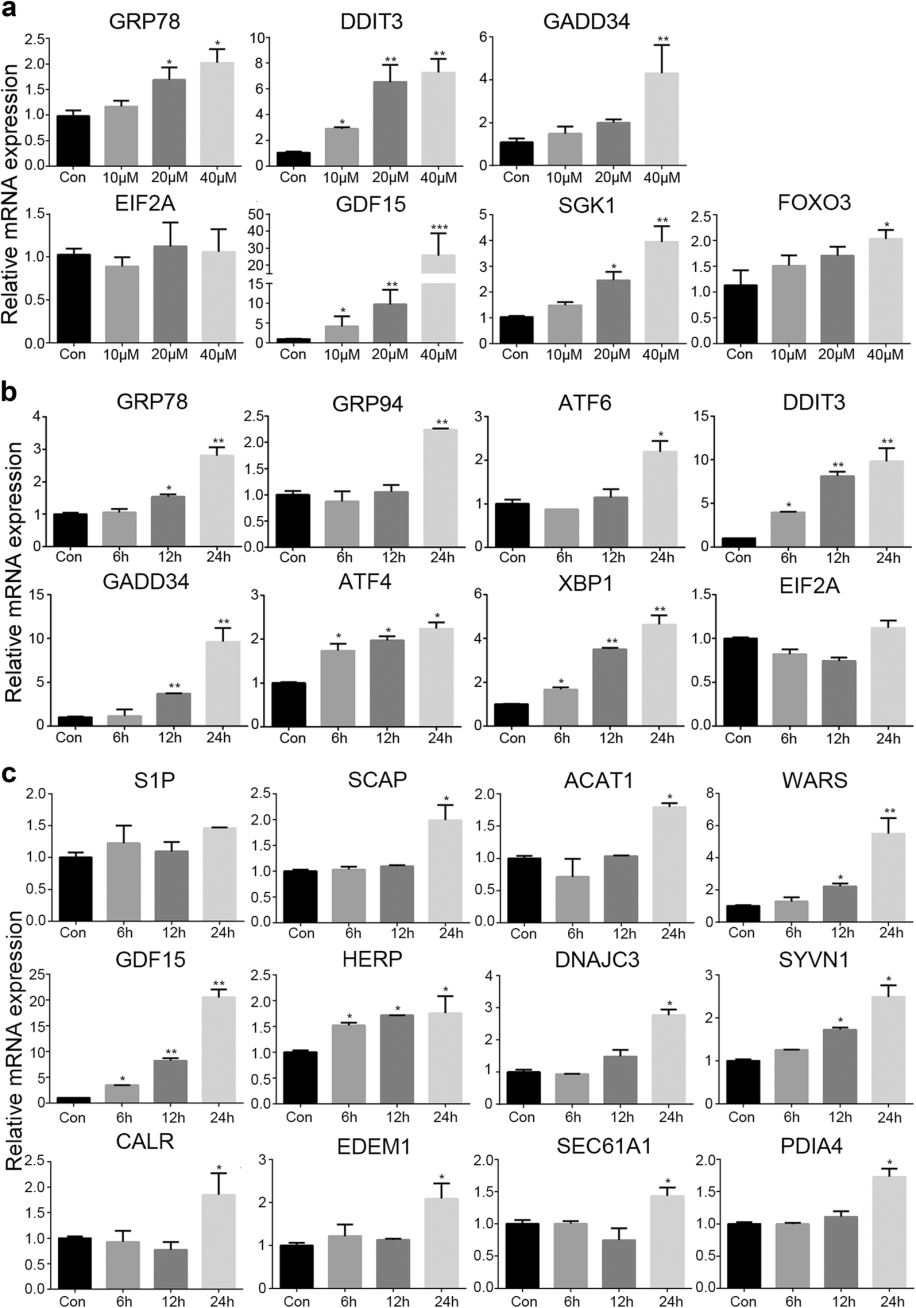
Effects of psoralen on gene expression of ER-stress. **a** The mRNA levels of key gene in ER-stress by different doses of psoralen treatment for 48 h. **b** The mRNA levels of key gene in ER-stress by 40Μm psoralen for 6 h, 12 h and 24 h. **c** The mRNA levels of ER functional gene by 40Μm psoralen for 6 h, 12 h and 24 h. Values are mean ± SD (n = 3) and * is means compared to the Con group, *p < 0.05; **p < 0.01; ***p < 0.001

To investigate the effects of psoralen on the key gene expression of ER-stress, we treated SMMC7721 with different doses and time duration. Compared with the control group, GRP78 and SGK1 mRNA expression were increased significantly in the treatment with 20 μM and 40 μM psoralen for 48 h (P < 0.05), DDIT3 and GDF15 mRNA levels were dose-dependent increased (P < 0.01), and relative mRNA expression of GADD34 and FOXO3 were also increased by 40 μM psoralen (P < 0.05). However, different doses of psoralen showed little influence on EIF2A mRNA expression (Fig. 6a). Next, we detected the mRNA levels with the treatment of 40 μM psoralen for 6 h, 12 h and 24 h. Compared with the control group, DDIT3, XBP1, and ATF4 mRNA expression were time-dependent increased (P < 0.05), GRP78 and GADD34 mRNA expression were increased significantly with the treatment for 12 h and 24 h (P < 0.05), and GRP94 and ATF6 mRNA levels were increased significantly after 24 h (P < 0.05) (Fig. 6b). We also detected some gene involved in other biological processes on ER. Compared with the control group, GDF15, HERP, WARS, SYVN1, SCAP, ACAT1, DNAJC3, CALR, EDEM1, SEC61A1, and PDIA4 mRNA expression were increased significantly by 40 μM psoralen after treatment for 24 h (P < 0.05) (Fig. 6c). However, psoralen treatment showed little effect on the mRNA levels of ER-stress gene in HepG2 (Additional file 1: Figure S1). These results showed that psoralen can induce unfolded proteins and cause endoplasmic reticulum stress in SMMC7721 hepatoma cells.

### The effects of psoralen on ER-stress related protein expression in SMMC7721

**Fig. 7:**
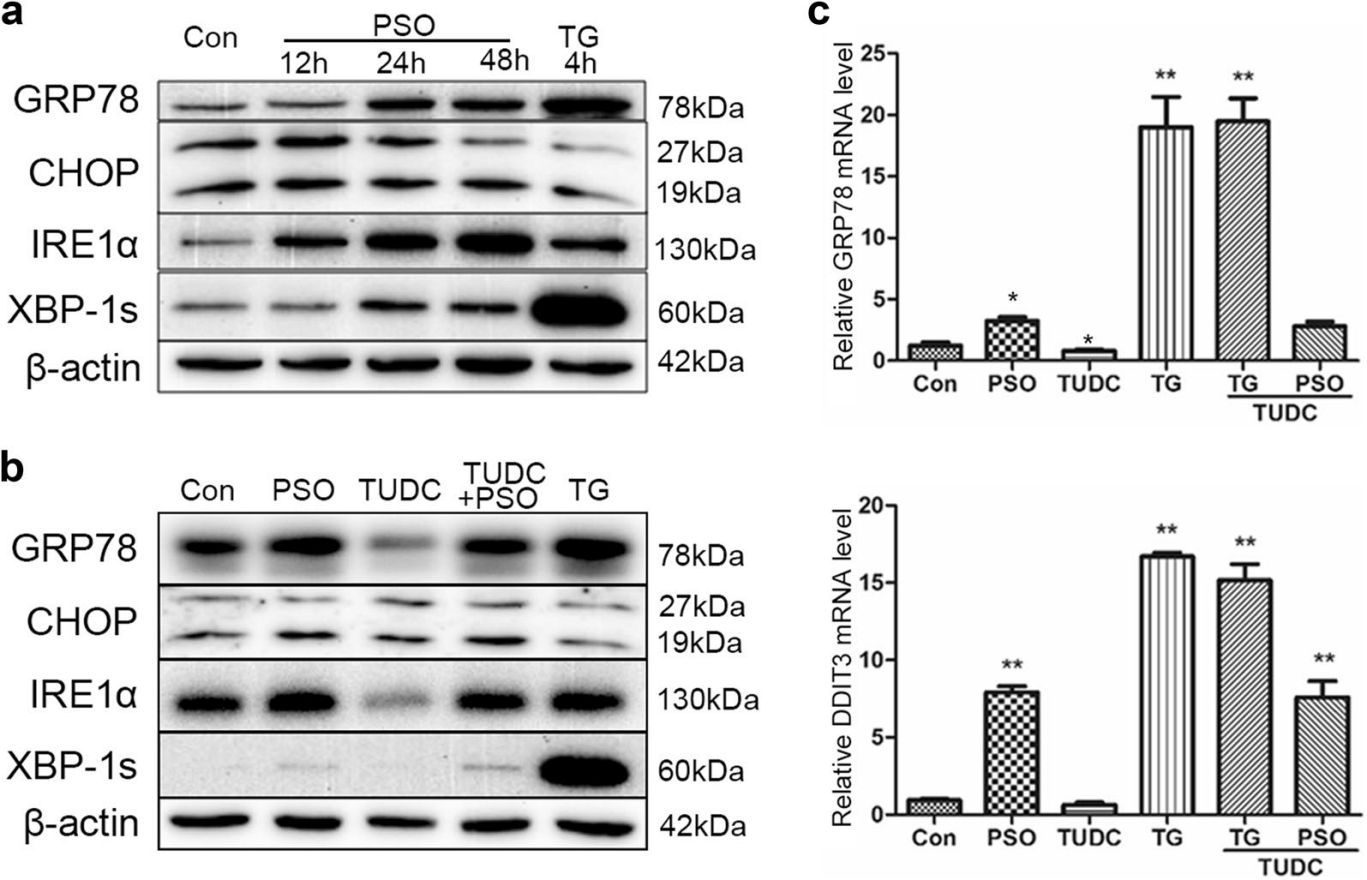
Effects of psoralen on ER-stress related protein expression in SMMC7721. **a** Western blot assays showing the effects of psoralen and thapsigargin on ER-stress related protein expression. **b** Western blot assays showing the effects of TUDC on the expression of ER-stress related protein induced by PSO. **c** The mRNA levels of GRP78 and DDIT3 with TUDC under PSO and TG treatment. Values are mean ± SD (n = 3) and * is means compared to the Con group, **P < 0.01

40 μM psoralen was used to explore the effect of psoralen on ER-stress related protein expression. Compared with the control group, GRP78 and XBP-1s protein expression were increased significantly after treatment for 24 h and 48 h (P < 0.05). And the level of IRE1α was also up-regulated time-dependently (P < 0.05). However, psoralen had no significant effect on the expression of CHOP protein. As a positive control drug, thapsigargin (TG) promoted the expression of GRP78 and XBP-1s protein only in 4 h (P < 0.05) (Fig. 7a). To further evaluate the effects on ER-stress, the ER-stress inhibitor TUDC was added with the treatment of psoralen. Western blot assay showed that TUDC could not inhibit the increased expression of GRP78, XBP-1s, IREα, and CHOP proteins induced by psoralen (Fig. 7b). Similarly, TUDC had little effects on the psoralen-induced up-regulation of GRP78 and DDIT3 mRNA expression (Fig. 7c). The results suggested that psoralen can induce the endoplasmic reticulum stress in SMMC7721 hepatoma cells.

## Discussion

Psoralen is extracted from dried fruits of *P. corylifolia* (L.) from *Leguminosae*, which shows an active effect including anti-tumor, photosensitivity, cardiovascular protection, anti-histamine and estrogen-like pharmacological effects [25–29]. In the traditional Chinese medicine, the property and flavor of *P. corylifolia* (L.) are spicy, bitter and warm, and it has warming kidney and enhancing yang, preventing asthma, to warm the kidney and the spleen and check diarrhea, and removing beverage effect [30], and *P. corylifolia* can be used as basic remedy to suppress tumor [13–15, 31, 32]. However, the roles of psoralen on the treatment of various tumors are not well understood. In our previous studies, we found that psoralen can inhibit the proliferation of Y1 adrenocortical tumor cells and AtT20 pituitary tumor cells. Based on these results, we explored the inhibition effects of psoralen on SMMC7721 human hepatoma cells for further study. Our results demonstrated that psoralen could significantly inhibit the proliferation of SMMC772 and induce apoptosis. In addition, we found that psoralen could induces cell cycle arrest at G1 phase and induce ER-stress to promote cell apoptosis, thus revealing the molecular mechanism of psoralen in anti-hepatoma effect in vitro.

As the malignant proliferation of hepatoma cells is an important characteristic of the biological behavior of tumor cells, the inhibition effect of psoralen on the proliferation of SMMC7721 hepatoma cells has an objective prospect of research and development. Similarly, psoralen can inhibit the proliferation of BGC-823 human gastric cancer cells and the half inhibitory rate (IC50) was 5.82 μg/mL [33]. Moreover, MG-63 osteogenic sarcoma cells were inhibited with 640 μmol/L psoralen treatment for 72 h, and the cell inhibition rate was 77.74%, and the cells became smaller, rounder, vacuoles and pseudopodia disappeared [34]. Although there are lots of studies about the pro-apoptotic function of psoralen, however, recent study demonstrated that psoralen could inhibit the apoptosis and increase the proliferation of osteoporotic osteoblasts by modulating IRE1–ASK1–JNK pathway [35].

Cell cycle disorder is an important feature of tumor cells, especially malignant transformation, so cyclin is an important target for the anti-cancer effect of psoralen. Among them, CyclinD1 and CyclinE1 play vital roles in the regulation of cell cycle, which are positive regulators of cyclin-dependent kinase (CDK). CyclinD1 associates with CDK4/6 and CyclinE1 binds to CDK2, which promote cells enter S phase from G1 phase, causing cell division and proliferation [36]. Once the proteins are over-expressed, the cell will shorten G1 phase and advance into S phase, causing continued proliferation and increasing the risk of cancer [37]. It has been reported that psoralen can form a complex with DNA in the form of non-covalent bond that cause DNA damage [38], arrest cells in G1 phase, and repair damaged DNA. Psoralen can arrest cells in G1 and G2 phases, and significantly reduce the proportion of S phase cells thereby inhibiting cell proliferation of MCF-7 human breast cancer cells [27]. However, another study reported that 10 μM and 30 μM psoralen can promote the progression of MCF-7 cells from G1 to S phase [39]. It suggested that the regulation of psoralen on cell cycle in different tumors was not the same.

Furtherly, psoralen can induce late apoptosis of SMMC7721 cells and show dose-effect relationship. According to recent studies, psoralen-induced apoptosis of K562 cells was observed under electron microscope [40]. In gastric cancer, early apoptosis of BGC-803 cells could be obvious after psoralen treatment for 48 h [41]. In this research, it suggests that psoralen has an exact apoptosis-inducing effect on SMMC7721 hepatoma cells, however, it is mainly not through the molecular mechanisms of the pro-apoptotic protein Bax and the apoptosis inhibitor protein Bcl-2.

As well known, apoptosis may be involved in endoplasmic reticulum stress. SMMC7721 cells were observed under transmission electron microscopy after 24 h of psoralen treatment, and we found that the endoplasmic reticulum was dilatated. Then the mRNA and the protein levels of endoplasmic reticulum-associated functional molecules were detected, which also indicated that psoralen could induce ER-stress. The endoplasmic reticulum is a membranous intracellular organelle, including rough endoplasmic reticulum and smooth endoplasmic reticulum. It is mainly involved in the processes like synthesis and secretion of membrane proteins, the correct folding of proteins, the storage of Ca^2+^, the synthesis and the metabolism of lipids and cholesterol [23]. Due to the influence of extrinsic or intrinsic factors, misfolded and unfolded proteins accumulate in the lumen of the endoplasmic reticulum as well as the disorder of the Ca^2+^ balance are known as ER-stress. The unfolded protein reaction in cells mainly involves three signaling pathways: double-strand RNA-activated protein kinase-like ER kinase (PERK) pathway, inositol-requiring enzyme 1 (IRE1) pathway, activating transcription factor 6 (ATF6) pathway. Under physiological conditions, PERK, IRE1 and ATF6 are all combined with glucose regulatory protein 78 (GRP78/Bip) in an inactive state [42]. Once ER-stress, a large number of unfolded or misfolded proteins will snatch GRP78 that originally binds to three kinds of responsive proteins, making them exposed and activated. At present, it has been found that GRP78 is closely related to the occurrence and development of liver cancer [43], breast cancer [44] and lung cancer [45]. The expression of GRP78 is increased when ER-stress occurs, which can promote the misfolded or unfolded protein to normal status, thereby reducing the endoplasmic reticulum load and maintaining the stability of the intracellular environment. Glucose regulatory protein 94 (GRP94) is mainly stored in the endoplasmic reticulum and is an important factor in the endoplasmic reticulum stress response. When stress occurs in the endoplasmic reticulum, GRP94 can induce the expression of C/EBP homologous protein, thereby down-regulating Bcl-2 and promoting apoptosis [46]. This study showed psoralen plays a clear role in promoting the expression of GRP78 and GRP94, which can induce unfolded protein reaction and cause apoptosis.

Because PERK is a type I endoplasmic reticulum transmembrane protein, its own activation of phosphorylation also results in phosphorylation of the catalytic substrate eIF2α. Phosphorylated eIF2α can inhibit the synthesis of protein and reduce the endoplasmic reticulum load in a short period of time, but long-term inhibition also leads to apoptosis. Furthermore, it can also promote the translation of activating transcription factor-4 (ATF-4) [47]. Overexpression of ATF4 can activate the CHOP apoptotic pathway, which can activate GADD34 to cause the damage of oxygen free radicals. ATF6 is a type II transmembrane protein of the endoplasmic reticulum, which is separated from GRP78 and can be activated by S1P and S2P cleavage during ER-stress. Activated ATF6 promotes transcription and expression of CHOP in the nucleus, thereby inhibiting Bcl-2 and causing apoptosis [48]. This results suggested that psoralen-induced endoplasmic reticulum stress mainly activates the IRE1 pathway and the ATF6 pathway. Once ER-stress occurs, phosphorylated eIF2α or IRE1 protein can activate GADD34 and ATF4. Activated GADD34 dephosphorylates p-eIF2α and restores protein synthesis [49]. Overexpression of GADD34 and ATF4 induces CHOP expression, inhibits Bcl-2 expression and causes damage of oxygen free radicals which can induce apoptosis. These results showed that psoralen increased GADD34 and ATF4 genes expression, which participating in endoplasmic reticulum stress and promoting apoptosis.

C/EBP homologous protein is known as DDIT3, which is in a low expression in physiological state, can activate apoptotic C/EBP homologous protein. During ER-stress, the PERK, IRE1 and ATF6 signaling pathways can promote CHOP expression. However, high expression of CHOP leads to the loss of Ca^2+^ in the endoplasmic reticulum, which increases mitochondrial permeability and induces apoptosis [50]. On the other hand, it also reduces the expression of anti-apoptotic proteins and increases the expression of pro-apoptotic proteins, finally activating cellular mitochondrial apoptosis pathway [51]. This study found that psoralen can promote significantly DDIT3 gene expression, but there is no significant effect on the CHOP protein level. Combined with the expression of apoptosis-related proteins Bcl-2 and Bax, it is speculated that psoralen may cause changes in Ca^2+^ concentration in the endoplasmic reticulum through highly expressed DDIT3 which triggers apoptosis.

In general, for short-term ER-stress, cells can maintain the stability of the intracellular environment by enhancing the ability of protein folding, inhibiting protein synthesis and translation, and accelerating protein degradation. They are self-protective and belong to normal physiological phenomena. When ER-stress continues, a large amount of unfolded or misfolded protein will accumulate, which will induce unfolded protein response (UPR) and cause apoptosis. This study indicated that psoralen could cause a significant induction of ER-stress in dose- and time-effect relationship, which causing apoptosis of hepatoma cells.

In addition, psoralen might regulate some physiological process in HCC by increased relevance mRNA levels on endoplasmic reticulum. Growth differentiation factor 15 (GDF15) is a divergent member of the BMP-subfamily of the TGF-β superfamily, which could inhibit the proliferation, migration and invasion, while promoting apoptosis of A549 cells [52]. Induction of ER stress leads to upregulation of several genes such as WARS (tryptophanyl-tRNA synthetase), HERP (homocysteine-inducible ER protein with ubiquitin like domain 1), DNAJC3 (also called P58IPK), ER degradation-enhancing alpha-mannosidase-like 1 (EDEM1) and leads to caspase activation, release of mitochondrial intermembrane proteins and dissipation of mitochondrial transmembrane potential (ΔΨm) [53]. Synovial apoptosis inhibitor 1 (SYVN1), an ER-associated degradation (ERAD) E3 ubiquitin ligase, could inhibit the breast cancer cell growth and metastasis through the miR-96-5p/SYVN1 axis [54]. Site 1 protease (S1P), SREBP cleavage-activating protein (SCAP) and acetyl-CoA acetyltransferase 1 (ACAT1) were involved in the lipid metabolism [55]. ER chaperone calreticulin (CALR), protein transport protein Sec61 subunit alpha isoform 1 (SEC61A1), protein disulfide-isomerase A4 (PDIA4) were up-regulated in the ER stress response for binding to misfolded proteins [56–58]. Besides, it is worth noting that TUDC, an ER-stress inhibitor, did not inhibit the expression of GRP78, DDIT3, XBP-1s, IREα, and CHOP, which were increased expression by psoralen. It suggested that psoralen-induced SMMC7721 cells have a strong ER stress response, and the damage to cells is irreversible and is not easily corrected by ER-stress inhibitor. It suggested that the psoralen-induced ER-stress promotes apoptosis of SMMC7721 hepatoma cells with specific characteristics.

## Conclusion

Psoralen inhibits proliferation of SMMC7721 cells by arresting cells at G1 phase. Moreover, it can also cause the endoplasmic reticulum expansion and dysfunction, so as to induce the ER-stress persistently, leading to the apoptosis of hepatoma cells. The study enriched the pharmacological mechanism of psoralen in the anti-hepatoma effect.

## Methods

### Materials

Psoralen (with the chemical structure illustrated in Fig. 1) was obtained from the Shanghai Institute for Food and Drug Control (NO. 110739, Shanghai, China; purity: more than 98% HPLC) and dissolved in dimethyl sulfoxide (DMSO, ST038, Biyotime, China). The final concentration of DMSO was 0.1% in all psoralen groups and had no effect on cell viability. Thapsigargin was obtained from Sigma-Aldrich (T9033, St. Louis, MO, USA), TUDC was purchased Selleck Chemicals (S3654, Houston, TX, USA).

DMSO and 3-(4,5-dimethylthiazol-2-yl)-2,5-diphenyltetrazolium bromide (MTT) were obtained from Sigma-Aldrich (M2128, St. Louis, MO, USA). Fetal bovine serum and RIPM1640 were purchased from Gibco (Grand Island, NY, USA), penicillin and streptomycin and 0.25% Trypsin (0.02% EDTA) were purchased from Hyclone (Marlborough, MA, USA). Trizol was obtained from Invitrogen Life Technologies (Grand Island, NY, USA), PrimeScript^®^RT reagent Kit, SYBR^®^ Premix Ex Taq™ (Tli RNaseH Plus) II were purchased from TaKaRa Bio Inc (Otsu, Japan). RIPA lysis buffer, BCA kit, IgG(H + L) and IgG(H + L) second antibodies were purchased from Beyotime Biotechnology (Shanghai, China), Annexin V-FITC Apoptosis Analysis Kit and PI/RNase Staining Solution were purchased from Sungen Biotech (Tianjing, China). CyclinD1 (ab134175), CyclinE1 (ab33911), CDK4 (ab68266), Bax (ab182733) and Bcl-2 (ab182858) antibodies were purchased from Abcam (Cambridge, MA, USA). GRP78 (3177s), GRP94 (2104s), IRE1α (3294s), XBP-1S (12782s), PERK (3192s) and p-eIF2α (9721s) antibodies were purchased from Cell signaling technology. β-actin (A5441) and p27 (SAB4500067) antibodies were purchased from Sigma-Aldrich (St. Louis, MO, USA). CHOP (NBP2-13172) antibody was purchased from Novus Biologicals (Littleton, CO, USA).

### Cell culture and treatment

The human hepatoma SMMC7721 cells were cultured in RIPM1640 medium containing 1% penicillin–streptomycin and 10% FBS. According to different experimental purposes, drug treatment was as follows: SMMC7721 cells were plated at a density of 5000 cells/well in 96-well plates with 24 h incubation, and treated with 10–80 μM psoralen for 24 h, 48 h and 72 h, then the proliferation of cells was detected by MTT assay. The cells were plated at a density of 1 × 10^4^ cells/well in 12-well plates with 24 h incubation, and treated with 40 μM psoralen and 0.1 μM thapsigargin for 12 h, 24 h and 48 h, then the cell morphologic change was observed by inverted microscope. The cells were plated into 6-well plates at a density of 1 × 10^5^ cells per well in 6-well plates with 24 h incubation, and treated with 40 μM psoralen and 0.1 μM thapsigargin for 24 h, then the ultrastructure of cells was observed by transmission electron microscope; or the cells were treated with 10–50 μM psoralen for 48 h, then the cell apoptosis and cell cycle were tested by flow cytometry; or the cells were treated with 40 μM psoralen for 6 h, 12 h, 24 h or 48 h, then the expression of mRNA and protein expression were detected by RT-qPCR or Western blotting. Additionally, 0.1% DMSO was as a control group in all experiments.

### MTT assay

Cell viability was tested using the MTT assay according to the manufacturer’s instructions. SMMC7721 cells were plated into 96-well plates at a density of 5000 cells/well for 24 h. Following treatment with psoralen for 24 h to 72 h, the medium was removed and cells were washed with PBS. MTT (0.5 mg/mL) was then added to each well and the mixture was incubated for 4 h at 37 °C. MTT reagent was then replaced with DMSO (150 μL per well) to dissolve formazan crystals. After the mixture was shaken at room temperature for 10 min, absorbance was measured at 490 nm using a microplate reader (Bio-Tek, Winooski, VT, USA).

### Routine transmission electron microscopy (TEM) of adherent cells

SMMC7721 cells were plated at a density of 1 × 10^5^ cells/well in 6-well plates and treated with 40 μM psoralen or 0.1 μM Thapsigargin for 24 h. Cells were fixed in situ in 2.5 % glutaraldehyde in 0.05 M phosphate buffer (pH 7.4) for 2 h at 4 °C, then cells were scraped down and to centrifuge for 15 min in 2000 rpm, then discarding the supernatant and resuspending in the 2.5% glutaraldehyde buffer. After fixation, the samples were post-fixed in 1% OsO_4_ for 4 h and dehydrated in increasing concentrations of ethanol and propylene oxide series. Subsequently, the samples were embedded in Epon 618 resin and polymerized for 48 h at 60 °C. Then, the ultrathin sections (90–100 nM) samples were mounted on the copper grids and were stained with 0.5% aqueous uranyl acetate and lead citrate. Subsequently, the grids were air-dried and examined under a transmission electron microscope (Philips Tecnai-12, Netherlands) at 25 kV.

### Flow cytometry for cell cycle and apoptosis

SMMC7721 cells were plated into 6-well plates at a density of 1 × 10^5^ cells per well in 6-well plates, after 24 h incubation at 37 °C, cells were treated with 40 μM psoralen for 48 h. Cycle arrest and apoptotic cells were detected by flow cytometric analysis. After the treatment, cells were collected by trypsinization and washed twice with PBS. For cell cycle assay, the collected cells were stained with propidium iodide (PI) (Sanjian, Tianjin, China). Cellular apoptosis was determined with Annexin V-FITC and PI using FITC Apoptosis Detection kit (Sanjian, Tianjin, China). Stained cells were assessed by BD FACSalibur flow cytometry (Franklin Lakes, NJ, USA), and the data were analyzed by FlowJo software (TreeStar, Ashland, OR, USA).

### Quantitative real-time reverse-transcription PCR (qRT-PCR)

Total RNA was extracted using TRIzol (Invitrogen) according to protocols from the manufacturers. The purity and integrity of the RNA were checked spectroscopically using a NanoDrop 2000/c spectrophotometer (Thermo). Then, for each sample, 2 μg RNA was reverse transcribed to obtain the cDNA template using PrimeScript^®^RT reagent Kit (TaKaRa). Each cDNA sample was diluted 5 times for qRT-PCR amplification; qRT-PCR was performed using the fluorescent dye SYBR^®^ Premix Ex Taq™ (Tli RNaseH PlusII, TaKaRa) with a 7500 Fast Real-Time PCR System. Amplification was performed with the following fast time course: 95 °C 30 s, 95 °C 5 s, 65 °C for 30 s for 40 cycles. Relative mRNA expression values were determined by the 2−ΔΔCt method using human β-actin as the normalization control.

### Western blotting

SMMC7721 cells were lysed with RIPA lysis buffer (Beyotime, Haimen, China) supplemented with PMSF. The proteins were separated through 10% SDS-PAGE gels electrophoresis, and transferred to polyvinylidene difluoride membranes, and incubated in block buffer and then incubated with primary anti-bodies, including Bax (1:2000), Bcl-2 (1:2000), CDK4 (1:1000), CyclinD1 (1:30,000), CyclinE1 (1:1000), P27 antibody (1:1000), GRP78 (1:1000), CHOP (1:1000), IRE1α (1:1000), XBP-1S (1:1000), and β-actin antibody (1:20,000), followed by incubation with horseradish peroxidase (HRP)-conjugated secondary antibody. Protein expression was visualized by enhanced chemiluminescence assay and signal Signals were detected using a Fluorchem E system (Protein-Simple, USA).

### Statistical analysis

Data were expressed as the mean ± SD. Significant differences were accepted at the 0.05 level of probability and were statistically determined with ANOVA followed by a Newman–Keuls post hoc test using GraphPad Prism6 (San Diego, CA).

## Additional file

**Additional file 1: Figure S1.** Effects of psoralen on gene expression of ER-stress in HepG2. The mRNA levels of key gene in ER-stress in the dose of 40 μM psoralen or 0.1 μM thapsigargin for 24 h. Values are means ± SD (n = 3) and * is means compared to the Con group, **, p < 0.01; ***, p < 0.001.

## Data Availability

All data generated or analysed during this study are included in this published article.

## Abbreviations

PSO: psoralen
HCC: hepatocellular carcinoma
ER: endoplasmic reticulum
TG: thapsigargin
PERK: protein kinase R-like ER kinase
ATF6: activating transcription factor 6
IRE1: inositol-requiring enzyme 1
GRP78: glucose-regulated protein of 78
UPR: unfolded protein response
CHOP: C/EBP homologous protein
XBP1: X-box binding protein 1
TUDC: tauroursodeoxycholic acid
qRT-PCR: quantitative real-time PCR

## References

[1] Torre LA, Bray F, Siegel RL, Ferlay J, Lortet-Tieulent J, Jemal A (2015). Global cancer statistics, 2012. CA Cancer J Clin.

[2] Siegel RL, Miller KD, Jemal A (2018). Cancer statistics, 2018. CA Cancer J Clin.

[3] Gish RG, Porta C, Lazar L, Ruff P, Feld R, Croitoru A, Feun L, Jeziorski K, Leighton J, Gallo J (2007). Phase III randomized controlled trial comparing the survival of patients with unresectable hepatocellular carcinoma treated with nolatrexed or doxorubicin. J Clin Oncol.

[4] Llovet JM, Ricci S, Mazzaferro V, Hilgard P, Gane E, Blanc JF, de Oliveira AC, Santoro A, Raoul JL, Forner A (2008). Sorafenib in advanced hepatocellular carcinoma. N Engl J Med.

[5] Jiang HL, Jin JZ, Wu D, Xu D, Lin GF, Yu H, Ma DY, Liang J (2013). Celastrol exerts synergistic effects with PHA-665752 and inhibits tumor growth of c-Met-deficient hepatocellular carcinoma in vivo. Mol Biol Rep.

[6] Jiang Z, Xiong J (2014). Induction of apoptosis in human hepatocarcinoma SMMC-7721 cells in vitro by psoralen from *Psoralea corylifolia*. Cell Biochem Biophys.

[7] Li S, Dong P, Wang J, Zhang J, Gu J, Wu X, Wu W, Fei X, Zhang Z, Wang Y (2010). Icariin, a natural flavonol glycoside, induces apoptosis in human hepatoma SMMC-7721 cells via a ROS/JNK-dependent mitochondrial pathway. Cancer Lett.

[8] Lee KY, Lee SK (1996). Ginsenoside-Rg1 positively regulates cyclin E-dependent kinase activity in human hepatoma SK-HEP-1 cells. Biochem Mol Biol Int.

[9] Zeng XL, Tu ZG (2004). Effect of telomerase on ginsenoside Rh2-induced differentiation of hepatocarcinoma cell line SMMC-7721. Chin J Cancer.

[10] Zhang L, Jiang G, Yao F, He Y, Liang G, Zhang Y, Hu B, Wu Y, Li Y, Liu H (2012). Growth inhibition and apoptosis induced by osthole, a natural coumarin, in hepatocellular carcinoma. PLoS ONE.

[11] Ou X, Chen Y, Cheng X, Zhang X, He Q (2014). Potentiation of resveratrol-induced apoptosis by matrine in human hepatoma HepG2 cells. Oncol Rep.

[12] Koul B, Taak P, Kumar A, Kumar A, Sanyal I (2018). Genus *Psoralea*: a review of the traditional and modern uses, phytochemistry and pharmacology. J Ethnopharmacol.

[13] Liu S, Zhou X, Wu J. Anti-tumor traditional Chinese medicine and preparation method thereof. In: vol. CN101961476A; 2010. CN101961476A.

[14] W. X, Dai L, Lin Y, Wang Z, Shang Q, Sun Y. Antitumor drugs and their preparation methods. In: vol. CN101007110; 2006. CN101007110.

[15] Zhao H. Traditional Chinese medicine for treating thyroid tumor. In: vol. CN105435048A; 2015. CN105435048A.

[16] Zhang Y, Wang Q, Wang ZX, Bi YN, Yuan XM, Song L, Jiang MM, Sun LK, Zhou K (2018). A study of NMR-based hepatic and serum metabolomics in a liver injury sprague-dawley rat model induced by Psoralen. Chem Res Toxicol.

[17] Wang Y, Hong C, Zhou C, Xu D, Qu HB (2011). Screening antitumor compounds psoralen and isopsoralen from *Psoralea corylifolia* L. seeds. Evid-Based Complement Altern Med.

[18] Liang L. Comparative study on the effects of the main components of traditional Chinese medicine on the regulation of endocrine function in mice. *master.* SHUTCM: SHUTCM; 2017.

[19] Jiang J, Wang X, Cheng K, Zhao W, Hua Y, Xu C, Yang Z (2016). Psoralen reverses the P-glycoprotein-mediated multidrug resistance in human breast cancer MCF-7/ADR cells. Mol Med Rep.

[20] Wu C, Sun Z, Ye Y, Han X, Song X, Liu S (2013). Psoralen inhibits bone metastasis of breast cancer in mice. Fitoterapia.

[21] Guo BF, Liu S, Ye YY, Han XH (2011). Inhibitory effects of osthole, psoralen and aconitine on invasive activities of breast cancer MDA-MB-231BO cell line and the mechanisms. Chin J Integr Med.

[22] Sheng L, Wu CY, Chen XF (2011). Inhibitory acting mechanism of psoralen–osthole on bone metastasis of breast cancer—an expatiation viewing from OPG/RANKL/RANK system. Chin J Integr Tradit West Med.

[23] Cubillos-Ruiz JR, Bettigole SE, Glimcher LH (2017). Tumorigenic and immunosuppressive effects of endoplasmic reticulum stress in cancer. Cell.

[24] Almanza A, Carlesso A, Chintha C, Creedican S, Doultsinos D, Leuzzi B, Luis A, McCarthy N, Montibeller L, More S (2018). Endoplasmic reticulum stress signalling—from basic mechanisms to clinical applications. FEBS J.

[25] Grundmann-Kollmann M, Tegeder I, Ochsendorf FR, Zollner TM, Ludwig R, Kaufmann R, Podda M (2001). Kinetics and dose–response of photosensitivity in cream psoralen plus ultraviolet A photochemotherapy: comparative in vivo studies after topical application of three standard preparations. Br J Dermatol.

[26] Coimbra S, Oliveira H, Neuparth MJ, Figueiredo A, Rocha-Pereira P, Santos-Silva A (2014). Inflammatory markers of cardiovascular disease risk in Portuguese psoriatic patients: relation with narrow-band ultraviolet B and psoralen plus ultraviolet A. Int J Dermatol.

[27] Wang X, Cheng K, Han Y, Zhang G, Dong J, Cui Y, Yang Z (2016). Effects of psoralen as an anti-tumor agent in human breast cancer MCF-7/ADR cells. Biol Pharm Bull.

[28] Bishnoi A, Parsad D, Vinay K, Kumaran MS (2017). Phototherapy using narrowband ultraviolet B and psoralen plus ultraviolet A is beneficial in steroid-dependent antihistamine-refractory chronic urticaria: a randomized, prospective observer-blinded comparative study. Br J Dermatol.

[29] Mar W, Je KH, Seo EK (2001). Cytotoxic constituents of *Psoralea corylifolia*. Arch Pharm Res.

[30] Wang S. Survey of pharmacological effects of psoralen. Lishizhen Med Mater Med Res. 2006;6(17).

[31] Zhang B, Zhang X, Zhao J (2017). Research status of semen *Psoraleae* anti-tumor effect. China Foreign Med Treat.

[32] Latha PG, Evans DA, Panikkar KR, Jayavardhanan KK (2000). Immunomodulatory and antitumour properties of *Psoralea corylifolia* seeds. Fitoterapia.

[33] Guo J, Wu H, Weng X, Yan J, Bi K (2003). Studies on extraction and isolation of active constituents from *Psoralen corylifolia* L. and the antitumor effect of the constituents in vitro. J Chin Med Mater.

[34] Lu Y, Yao L, Meng Q, Dong X, Ma L (2010). Effect of psoralen on proliferation and apoptosis of human osteosarcoma MG-63 cells. J Xinjiang Med Univ.

[35] Chen S, Wang Y, Yang Y, Xiang T, Liu J, Zhou H, Wu X (2017). Psoralen inhibited apoptosis of osteoporotic osteoblasts by modulating IRE1–ASK1–JNK pathway. BioMed Res Int.

[36] Ingham M, Schwartz GK (2017). Cell-cycle therapeutics come of age. J Clin Oncol.

[37] Mills CC, Kolb EA, Sampson VB (2017). Recent advances of cell-cycle inhibitor therapies for pediatric cancer. Cancer Res.

[38] Wu J, Wei W, Yuan Y (2011). Advances in studies on the chemical constituents and pharmacological effects of psoraleae. Drug Eval Res.

[39] Shen L, Zhao P, Niu J, Wang J (2007). Effect of psoralen on proliferation of human breast cancer cells. Chin Pharm Bull.

[40] Zhang DJ, Huang SL, Chen NN, Xiang Y, Yang PM, Zhao JY (2005). Growth-inhibiting effect of psoralen plus ultraviolet-A light therapy on K562 cells. J Chin Integr Med.

[41] Yan W, Zhang Y, Liu J, Wu J, Zhou X, Liu S, Yang J, Yu X (2015). Effect of psoralen on proliferation and apoptosis of gastric cancer cell line BGC-803 and its mechanism. Jilin J Tradit Chin Med.

[42] Patil C, Walter P (2001). Intracellular signaling from the endoplasmic reticulum to the nucleus: the unfolded protein response in yeast and mammals. Curr Opin Cell Biol.

[43] Luo C, Xiong H, Chen L, Liu X, Zou S, Guan J, Wang K (2018). GRP78 promotes hepatocellular carcinoma proliferation by increasing FAT10 expression through the NF-kappaB pathway. Exp Cell Res.

[44] Cai Y, Zheng Y, Gu J, Wang S, Wang N, Yang B, Zhang F, Wang D, Fu W, Wang Z (2018). Betulinic acid chemosensitizes breast cancer by triggering ER stress-mediated apoptosis by directly targeting GRP78. Cell Death Dis.

[45] Kwon D, Koh J, Kim S, Go H, Min HS, Kim YA, Kim DK, Jeon YK, Chung DH (2018). Overexpression of endoplasmic reticulum stress-related proteins, XBP1s and GRP78, predicts poor prognosis in pulmonary adenocarcinoma. Lung Cancer.

[46] Rosenbaum M, Andreani V, Kapoor T, Herp S, Flach H, Duchniewicz M, Grosschedl R (2014). MZB1 is a GRP94 cochaperone that enables proper immunoglobulin heavy chain biosynthesis upon ER stress. Genes Dev.

[47] Hetz C (2012). The unfolded protein response: controlling cell fate decisions under ER stress and beyond. Nat Rev Mol Cell Biol.

[48] Xu W, Lu X, Zheng J, Li T, Gao L, Lenahan C, Shao A, Zhang J, Yu J (2018). Melatonin protects against neuronal apoptosis via suppression of the ATF6/CHOP pathway in a rat model of intracerebral hemorrhage. Front Neurosci.

[49] Dalet A, Arguello RJ, Combes A, Spinelli L, Jaeger S, Fallet M, Vu Manh TP, Mendes A, Perego J, Reverendo M (2017). Protein synthesis inhibition and GADD34 control IFN-beta heterogeneous expression in response to dsRNA. EMBO J.

[50] Copanaki E, Schurmann T, Eckert A, Leuner K, Muller WE, Prehn JH, Kogel D (2007). The amyloid precursor protein potentiates CHOP induction and cell death in response to ER Ca^2+^ depletion. Biochim Biophys Acta.

[51] Szegezdi E, Macdonald DC, Ni Chonghaile T, Gupta S, Samali A (2009). Bcl-2 family on guard at the ER. Am J Physiol Cell Physiol.

[52] Duan L, Pang HL, Chen WJ, Shen WW, Cao PP, Wang SM, Liu LL, Zhang HL (2019). The role of GDF15 in bone metastasis of lung adenocarcinoma cells. Oncol Rep.

[53] Gupta S, Cuffe L, Szegezdi E, Logue SE, Neary C, Healy S, Samali A (2010). Mechanisms of ER stress-mediated mitochondrial membrane permeabilization. Int J Cell Biol.

[54] Gao Z, Wang H, Li H, Li M, Wang J, Zhang W, Liang X, Su D, Tang J (2018). Long non-coding RNA CASC2 inhibits breast cancer cell growth and metastasis through the regulation of the miR-96-5p/SYVN1 pathway. Int J Oncol.

[55] Basseri S, Austin RC (2012). Endoplasmic reticulum stress and lipid metabolism: mechanisms and therapeutic potential. Biochem Res Int.

[56] Winship AL, Sorby K, Correia J, Rainczuk A, Yap J, Dimitriadis E (2017). Interleukin-11 up-regulates endoplasmic reticulum stress induced target, PDIA4 in human first trimester placenta and in vivo in mice. Placenta.

[57] Jo SH, Choi JA, Lim YJ, Lee J, Cho SN, Oh SM, Go D, Kim SH, Song CH (2017). Calreticulin modulates the intracellular survival of mycobacteria by regulating ER-stress-mediated apoptosis. Oncotarget.

[58] Sadighi Akha AA, Harper JM, Salmon AB, Schroeder BA, Tyra HM, Rutkowski DT, Miller RA (2011). Heightened induction of proapoptotic signals in response to endoplasmic reticulum stress in primary fibroblasts from a mouse model of longevity. J Biol Chem.

