# Supplementary material for: Psoralen inhibits malignant proliferation and induces apoptosis through triggering endoplasmic reticulum stress in human SMMC7721 hepatoma cells
Source: Biol Res. 2019 Jul 5;52:34. doi: 10.1186/s40659-019-0241-8 (PMC6612100; doi:10.1186/s40659-019-0241-8)
Supplement: Supplementary file 1 — Additional file 1: Figure S1. Effects of psoralen on gene expression of ER-stress in HepG2. The mRNA levels of key gene in ER-stress in the dose of 40 μM psoralen or 0.1 μM thapsigargin for 24 h. Values are means ± SD (n = 3) and * is means compared to the Con group, **, p < 0.01; ***, p < 0.001. [file 40659_2019_241_MOESM1_ESM.docx]

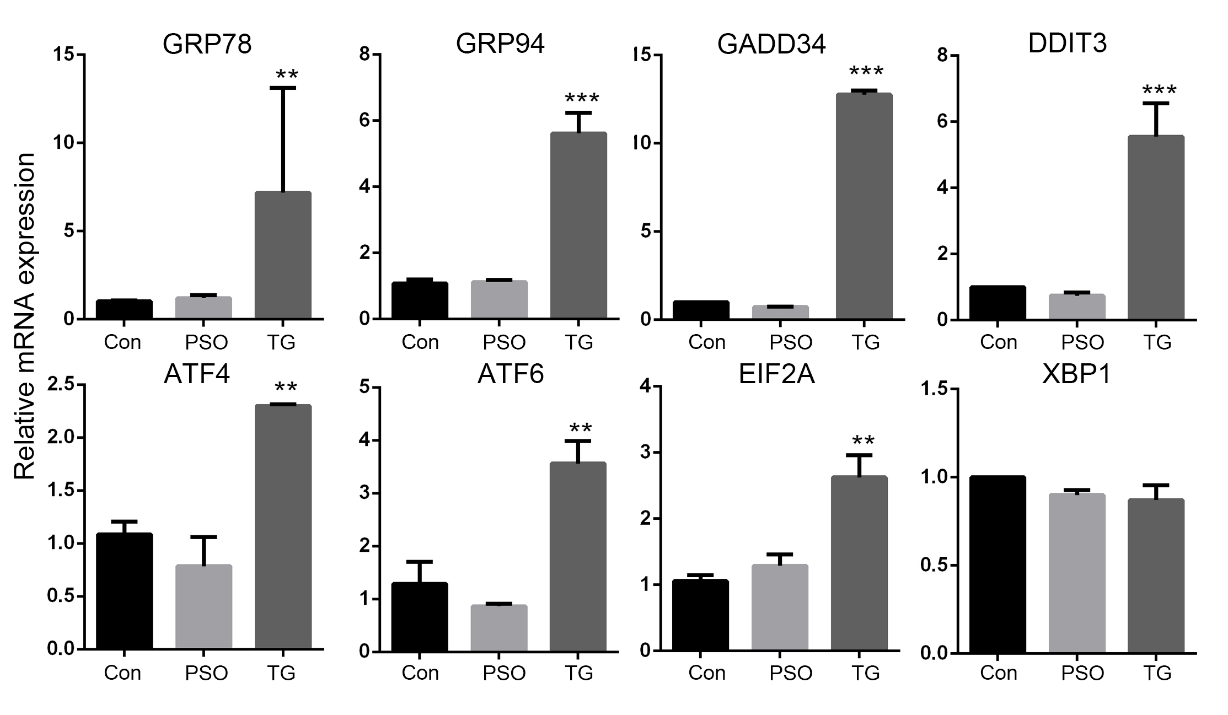


**Figure S1. Effects of psoralen on gene expression of ER-stress in HepG2**. The mRNA levels of key gene in ER-stress in the dose of 40μM psoralen or 0.1μM thapsigargin for 24h. Values are means ± SD (n=3) and * is means compared to the Con group, **, p<0.01; ***, p<0.001,
